# Ovarian reserve in women with endometriosis under total cystectomy compared to partial cystectomy: A randomized clinical trial

**DOI:** 10.18502/ijrm.v19i7.9472

**Published:** 2021-08-16

**Authors:** Atiye Javaheri, Samane Kabirpour Ashkezar, Maryam Eftekhar, Seiede Zahra Ghanadzade Tafti

**Affiliations:** Obstetrics and Gynecology Department, Faculty of Medicine, Shahid Sadoughi University of Medical Sciences, Yazd, Iran.

**Keywords:** Anti-Müllerian hormone, Endometriosis, Cystectomy, Ovarian reserve.

## Abstract

**Background:**

The standard procedure for ovarian endometriosis is laparoscopic excision of ovarian cysts and complete removal of the cyst capsule using the striping technique. Owing to the possibility of reducing ovarian reserve, and in some cases, the premature ovarian failure, the safety remains to be known.

**Objective:**

To compare the ovarian reserve in women with endometriosis who underwent total cystectomy with partial cystectomy.

**Materials and Methods:**

In this randomized clinical trial, 56 women with endometriosis who were referred to the Research and Clinical Center for Infertility and Shahid Sadoughi Hospital, Yazd, Iran between January and February 2020 were randomly assigned into two groups (n = 25/each); group I (total cystectomy) and group II (partial cystectomy). To assess the ovarian reserves, the anti-Müllerian hormone (AMH) level before and three months after surgery was measured and compared between the two groups.

**Results:**

No significant difference was observed in the AMH levels before and after surgery (p = 0.52, p = 0.32, respectively). However, the mean reduction of AMH in total cystectomy group was significantly higher than the partial cystectomy (p = 0.001).

**Conclusion:**

Cystectomy in women with endometriosis reduces ovarian reserve and can help maintain some ovarian reserve by performing partial instead of total cystectomy.

## 1. Introduction

Endometriosis is defined as the presence of endometrial tissue including glands and stroma outside the uterus. The most common regions for endometrial tissue implantation are pelvic viscera and peritoneum. Endometriosis ranges from a few small lesions on the pelvic components, that appear to be normal in other respects, to large ovarian endometriosis cysts that also affect the intestines, bladder, and ureter (1, 2). The disease is found in all racial groups, mainly in women of childbearing age, but has also been reported in adolescents and postmenopausal women who are being treated with alternative hormones. Although the estimates of endometriosis frequency vary considerably, the prevalence of the disease in the general population is reported to be 0.8–28.6% with an overall estimation of 4.4%, often associated with pelvic pain and infertility (3, 4).

The standard approach to ovarian endometriosis is to remove the cyst capsule by laparoscope using the striping technique (5). Although the safety of this technique remains unknown, it can reduce ovarian reserve and trigger cases of premature ovarian failure in infertile women (6).

Today, anti-Müllerian hormone (AMH) is considered as the best predictor of ovarian reserve (7). Antral perineal granulosa cells and small antral follicles produce AMH (8). Serum AMH concentration in women with a normal menstrual cycle is in the normal range and decrease with age so that it becomes indistinguishable after menopause (8, 9).

This hormone appears to be related to the number of antral follicles and can be measured at any time in the cycle (7, 9, 10). Bodies of evidence suggest that AMH levels often diminish after endometriosis removal surgery (6). Rie Ozaki and coworkers examined the factors affecting ovarian reserve in women with endometriosis after cystectomy and showed that there was no significant decrease in the AMH and ovarian reserve in the partial cystectomy group (4).

Therefore, this study was designed to investigate the ovarian reserve after total cystectomy and compare it with partial cystectomy in women with endometriosis.

## 2. Materials and Methods

This randomized clinical trial was performed on 56 women with endometriosis who were referred to the Research and Clinical Center for Infertility and Shahid Sadoughi Hospital, Yazd, Iran between January and February 2020. The sample size according to a previous study (2), with the confidence level of 95%, a power of 80%, the statistical significance level of 0.05, the difference in the mean AMH of 0.04 unit (which if observed, revealed a reduction in ovarian reserve), and attrition of 10% was determined to be 28 in each group.

The inclusion criteria were: women aged 20–40 yr, AMH > 1 ng/mL, and endometrioma size > 4 cm. Besides, women with pregnancy, menstrual bleeding disorders, history of any ovarian surgery altering pelvic anatomy, follicle-stimulating hormone (FSH) being at menopause level, suspected ovarian malignancies, taking oral contraceptive pills three months prior to surgery, and endocrine disorder including thyroid disease, hyperprolactinemia, or Cushing's syndrome were excluded.

The cyst's diameter was measured using vaginal ultrasound or an MRI scan, and in cases of bilateral endometriosis, the total size of the two cysts was measured. In addition, the AMH and cancer antigen 125 (CA125) concentration levels were measured by ELISA before surgery. Then, the participants were divided into two groups based on a table of random numbers; group I (total cystectomy) and group II (partial cystectomy). To reduce the difference between the surgical techniques as much as possible, all participants were operated on by one surgeon.

All women underwent general anesthesia and endotracheal intubation in a lithotomy status with Trendelenburg position at 30∘. CO${}_{2}$ gas was introduced with a 10-mm trocar through the open method and 10-mm Hg pressure and under the supervision of a 10-mm rigid laparoscope through the umbilical port. Two other 5-mm ports were created 2 cm above the ASIS and a uterine manipulator was inserted into the participants' uterus; then the aspirate cyst and the discharge and adhesion between the cyst and the pelvic wall were released. The cyst was then gently separated from the normal ovarian cortex using two traumatic forceps. In the partial cystectomy, the umbilical part of the ovary remained in place. In this method, like the total cystectomy, the internal surface of the cyst was separated from the ovary using two forceps; and this continued until either it began to bleed or the cleavage level, that is, the ovarian Hilus, was not distinctively clear; it was here that cystectomy was stopped, as there was the greatest risk of damage to primary and secondary follicles. Using the striping technique, 80–90% of the cyst was removed and the rest of the hemostasis was established by cauterization. Three months after the surgery, serum AMH levels were evaluated and compared between the two groups. The AMH reduction was also measured using the following formula:

 Reduction  level  of  AMH (%)= AMH  before  surgery −− AMH  after  surgery  AMH  before  surgery ×100

### Ethical considerations

The study proposal was reviewed and approved by the ethics committee of the School of Medicine, Shahid Sadoughi University of Medical Sciences, Yazd, Iran (Code: IR.SSU.MEDICINE.REC.1398.296) and registered at the Iranian Clinical Trials. In addition, a written informed consent was obtained from all participants before the initiation of the trial following a full explanation of the purpose and method of the study. Participants were also assured that all their information would be kept confidential and would only be used for research purposes.

### Statistical analysis

After encoding, the collected data were entered into SPSS v18 statistical software (Statistical Package for the Social Sciences, SPSS, Chicago, IL, USA). The mean, standard deviation, and relative frequency indices of the variables were calculated and compared using analytical tests including Student's *t* test and Chi-square test between the two groups. The significance level was considered to be 0.05.

## 3. Results

**Figure 1:**
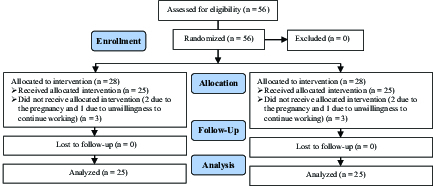
Consort flowchart of study participants.

In this study, 56 women with endometriosis were randomly assigned into two groups: total cystectomy and partial cystectomy (n = 28/each). During the study, three women from each group (two due to pregnancy and one due to unwillingness to continue working) were excluded from the study, and finally, the data of 25 participants were statistically analyzed and compared (Figure 1).

Participants in the two groups did not differ significantly in terms of demographic characteristics including age, endometrioma size, and CA125 level (Table I). Also, a comparison of AMH serum levels in the two groups showed no statistically significant difference before and after the surgery (p = 0.052 and p = 0.32, respectively), however, the mean decrease in AMH in both groups turned out to be significant (p = 0.001) (Table II).

**Table 1:** Demographic characteristics of the participants in two study groups (n = 25/each)


**Variables**	**Group I (Total cystectomy)**	**Group II (Partial cystectomy)**	p-value*
**Age (yr)**	29.88 ± 5.4	29.58 ± 5.7	0.80
**Endometrioma size (cm)**	5.52 ± 1.32	6.00 ± 1.35	0.21
**CA125 level (IU/ml)**	77.88 ± 32.4	70.54 ± 34.2	0.44
Data presented as Mean ± SD, *Student's *t* test			

**Table 2:** Comparison of mean AMH and its reduction rate in the two study groups before and after the surgery (n = 25/each)


**Variables**		**Group I (Total cystectomy)**	**Group II (Partial cystectomy)**	p-value*
**AMH level (ng/ml)**				
	**Before operation**	4.48 ± 2.62	4.52 ± 2.57	0.052
	**After operation**	2.75 ± 1.52	3.26 ± 1.62	0.32
**AMH reduction rate (%)**		36.39 ± 11.19	24.66 ± 12.11	0.001
All data presented as Mean ± SD, *Student's *t* test				

## 4. Discussion

This study aimed to compare the ovarian reserve through total and partial cystectomy in women with endometriosis. The results of the present study showed no significant difference in the AMH levels before and after surgery, but the mean reduction of AMH in total cystectomy group was significantly higher than the partial cystectomy (p = 0.001). This means that the reduction in ovarian reserve in total cystectomy was significantly higher than the partial cystectomy.

In 2016, Taniguchi and coworkers examined the rate of AMH changes, 6 months and 1 yr after laparoscopic cystectomy in 40 women with endometriosis and 16 women with benign ovarian tumors. Resultantly, they identified a remarkable reduction of AMH in the endometriosis group, especially in the age group > 35 yr compared to the non-endometriosis group (2). Also, Alborzi and coworkers assessed the serum levels of AMH and FSH before, 1 wk, and three and nine months after surgery in 193 women with endometrium who underwent laparoscopic cystectomy. They showed that after laparoscopic cystectomy for the endometrium, especially in elderly patients and those with bilateral cysts, AMH levels decreased and FSH levels increased (11). In the present study, we also observed a decrease in ovarian reserve and AMH levels after endometrioma cystectomy.

Furthermore, Ding and colleagues investigated the effect of laparoscopic cystectomy on ovarian reserve and the AMH concentration changes in bilateral endometriosis, unilateral endometriosis, and other unilateral ovarian cysts, at 1, 6, and 12 months after the operation. Their results indicated that the AMH concentration changes one month after surgery is significantly reduced in the bilateral endometrioma group. However, this difference was not significant compared to the 6 and 12 months following the surgery (5). Another study compared the effects of laparoscopic cystectomy with vaporization on AMH serum concentration in women with endometriosis. They showed that the laparoscopic cystectomy reduced AMH more than the other surgical procedures (3), thus being in line with the results of ours. Factors affecting ovarian reserve after endometriosis cystectomy were also investigated by Viganò and others. Their results showed that bilateral cystectomy had the greatest effect on AMH after surgery and no significant reduction in ovarian reserve was seen in the partial cystectomy (12). Donnez and colleagues also studied 52 women affected with endometriosis who underwent partial laparoscopic cystectomy surgery. By examining ovarian reserve six months after surgery, no significant difference was identified between these women and their age-matched group who did not undergo surgery (6). We also found similar results and showed a lower reduction of ovarian reserve in partial cystectomy compared with the total cystectomy group.

## 5. Conclusion

The results of the present study revealed that cystectomy in women with endometriosis reduces ovarian reserve and can help maintain some ovarian reserve by performing partial instead of total cystectomy especially in women with endometriosis who often suffer from infertility.

##  Conflict of Interest

The authors declare that they have no conflict of interest.
